# Effect of acupuncture for diminished ovarian reserve: study protocol for a randomized controlled trial

**DOI:** 10.1186/s13063-021-05684-w

**Published:** 2021-10-19

**Authors:** Huanfang Xu, Mingzhao Hao, Chensi Zheng, Huisheng Yang, Yaqian Yin, Li Yang, Yigong Fang

**Affiliations:** 1 Institute of Acupuncture and Moxibustion, China Academy of Chinese Medical Sciences, Beijing, China; 2 Acupuncture and Moxibustion Hospital of China Academy of Chinese Medical Sciences, Beijing, China; 3 Institute for History of Medcine and Medical Literature, China Academy of Chinese Medical Sciences, Beijing, China

**Keywords:** Diminished ovarian reserve, Acupuncture, Randomized controlled trial, Study protocol

## Abstract

**Background:**

Diminished ovarian reserve (DOR) is a condition in which the ovary loses its normal reproductive potential, compromising fertility. Although the prevalence and incidence of DOR is increasing, there are currently no effective treatments for this condition. Acupuncture has been reported as an alternative therapy for female infertility. The purpose of this study is to investigate the effect of acupuncture for women with DOR.

**Methods/design:**

In this randomized controlled trial, a total of 120 women with DOR will be randomly assigned to receive either acupuncture or sham acupuncture for 12 weeks. The primary outcome will be determined by the mean change from baseline in the antral follicle count (AFC) at week 12. Secondary outcomes include serum levels of FSH, LH, E2, and AMH, the length of menstrual cycle, and the score of Self-Rating Anxiety Scale (SAS).

**Discussion:**

This study is expected to investigate the effectiveness of acupuncture versus sham acupuncture in improving ovarian reserve for women with DOR.

**Trial registration:**

Acupuncture-Moxibustion Clinical Trial Registry ChiCTR1800014988. Registered on 6 February 2018

## Background

Decreased or diminished ovarian reserve (DOR) refers to women of reproductive age having regular menses whose response to ovarian stimulation is reduced in comparison to women of similar age [1]. Currently, there is no uniformly accepted definition of DOR. The term DOR may refer to three related but distinctly different outcomes: oocyte quality, oocyte quantity, and reproductive potential. Ten percent of women (totaling 275,000) in an infertility clinic of USA were diagnosed with DOR [2, 3]. Data from the US-based national Society for Assisted Reproductive Technology system showed 32% of in vitro fertilization (IVF) cycles (approximately 66,000 cycles) carried a diagnosis of DOR [4]. In China, the infertility rate of childbearing couples has increased from 3% 20 years ago to 15% at present [5]. Among them, infertility caused by DOR accounts for 10% of the total number of female infertility [6]. A significant upward trend in the prevalence of DOR was noted in patients up to 40 years of age [7, 8]. Without timely and effective interventions, women with DOR may experience ovarian atrophy within 1 to 6 years, and their disease progress to primary ovarian insufficiency (POI) simultaneously [9].

At present, few modalities are available for the treatment of DOR. The commonly used hormone replacement therapy (HRT) [10] is mainly prescribed for women with DOR without birth demand. HRT is effective for relieving low estrogen symptoms but with a high recurrence rate after drug discontinuation and does not improve ovarian function. An international survey [11] indicated DOR patients respond poorly to controlled ovarian stimulation (COS) resulting in retrieval of fewer oocytes, producing poorer quality embryos, and reduced implantation rates and pregnancy rates. Some studies suggested the addition of growth hormone [12] during ovarian stimulation could enhance the response of granulosa cells to gonadotropins and dehydroepiandrosterone [13] could improve ovarian function. However, there is insufficient evidence to support their correlation. Thus, effective treatment methods for DOR are still needed to improve female reproductive health and their quality of life.

Acupuncture has been used for the treatment of female infertility for thousands of years in China. A prospective observational study [14] reported significant decrease of follicle-stimulating hormone (FSH) and luteinizing hormone (LH) levels and increase of estradiol (E2) level among DOR patients treated with acupuncture. Similar results were observed in randomized controlled trials (RCTs) for women with POI [15], a condition showing a more serious decline of ovarian function than DOR. In recent years, acupuncture has gained worldwide popularity as an adjunct to assisted reproductive technology. A higher clinical pregnancy rate was reported in patients receiving acupuncture prior to and post-embryo transfer during IVF-ET cycles [16, 17]. In this trial, a prospective randomized sham-controlled study will be conducted to investigate the effect of acupuncture for women with DOR.

## Study design and methods

### Study design

This is a prospective, single-center, randomized, sham-controlled trial. Participants will be assigned to receive acupuncture or sham acupuncture in a 1:1 ratio. This study is in accordance with the principles of the Declaration of Helsinki and has been approved by the ethics committee of the Institute of Acupuncture and Moxibustion, China Academy of Chinese Medical Sciences (CACMS) (approval number: SC2017-12-22-1-1). Written informed consent will be obtained from each participant before enrolment.

**Fig. 1:**
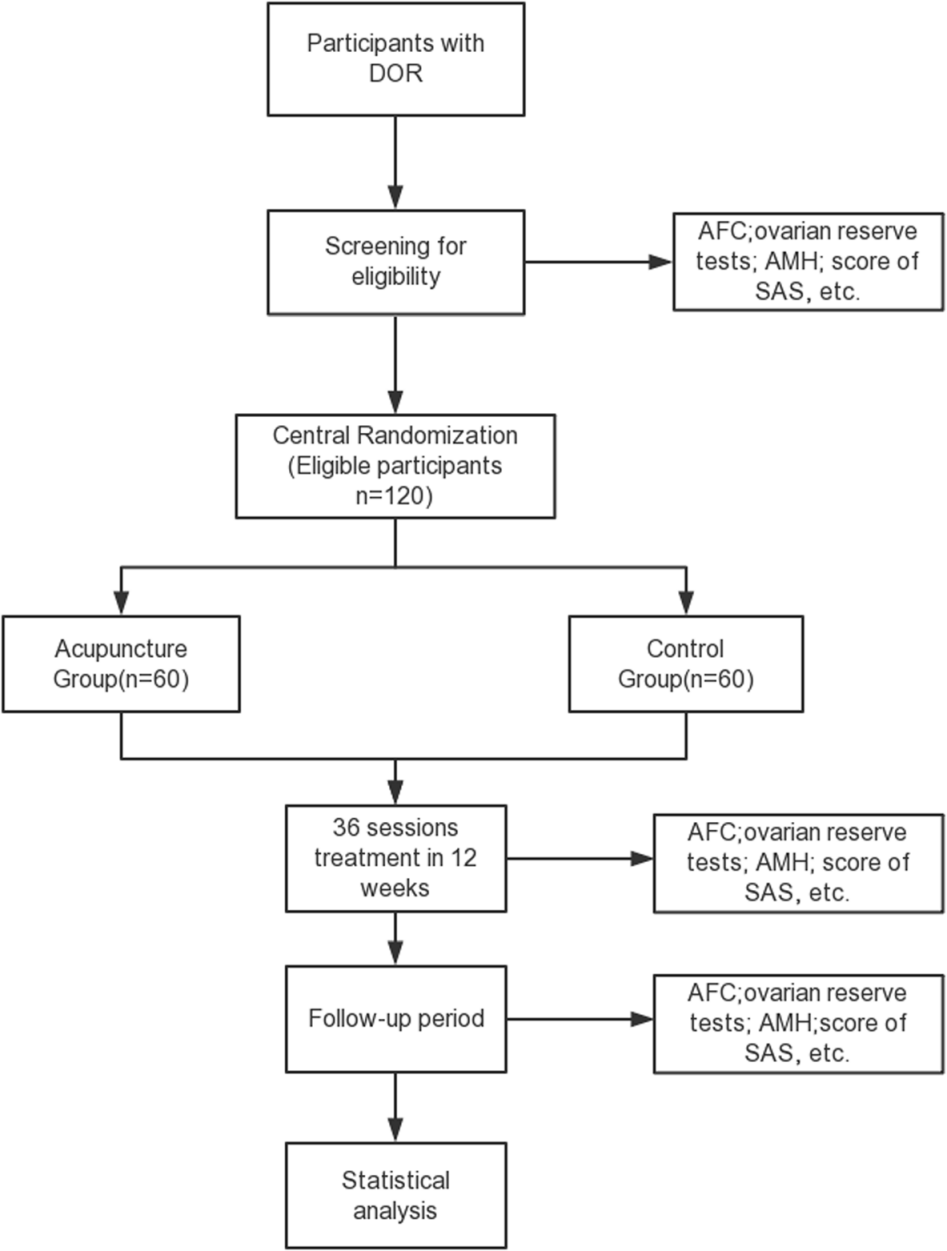
Trial flow chart

Total observation period will be 32 weeks, including an 8-week screening period (weeks − 8–0), a 12-week intervention period (weeks 0–12), and a 12-week follow-up period (weeks 13–24). Assessments will be conducted at baseline, week 12, and week 24. Flowchart and study design schedule are presented in Fig. 1 and Table 1, respectively.

**Table 1:** Study schedule of enrolment, intervention, and assessments

Time point	Screening period (data collection)	Intervention period		Follow-up period
	Weeks − 8 to 0	Week 1–week 11	Week12	Week 24
Enrolment				
Informed consent	×			
Eligibility screen	×			
Randomization	×			
Allocation	×			
Interventions				
Acupuncture group		×	×	
Control group		×	×	
Assessments				
AFC	×		×	×
Serum levels of FSH, LH, and E2	×		×	×
AMH	×		×	×
Length of menstrual cycle	×		×	×
SAS score	×		×	×
Safety				
Adverse events	×	×	×	×

*AFC* antral follicle count, *AMH* anti-Müllerian hormone, *FSH* follicle stimulating hormone, *LH* luteinizing hormone, *E2* estradiol, *SAS* Self-Rating Anxiety Scale

### Participants

A total of 120 patients with DOR will be recruited from outpatients of acupuncture and moxibustion hospital of CACMS via hospital poster, WeChat (a free popular social application in China) or network advertisement. Two staff members who are responsible for participant recruitment will conduct the consenting process in a separate clinic room in our hospital. Participants will be informed of details of the study which includes but is not limited to the objectives, interventions, potential benefits, and risks. Should the participants agree to join the study, they are required to submit a signed informed consent form.

### Inclusion criteria

Participants who meet the following inclusion criteria will be included: (1) female, aged 18–40 years; (2) meet any of the following three items in both tests with an interval of at least 4 weeks: (a) 10 IU/L < FSH < 20 IU/L, tested on days 2–4 of menstrual cycle; (b) anti-Müllerian hormone (AMH) < 1.1 ng/mL; (c) antral follicle count (AFC) < 5–7, tested on days 2–4 of menstrual cycle; and (4) voluntarily join the research and sign the informed consent.

### Exclusion criteria

Participants meeting any of the following criteria will be excluded: (1) polycystic ovary syndrome, hyperprolactinemia, hyperandrogen, hypothyroidism, and other endocrine diseases which may inhibit ovulation; (2) DOR caused by iatrogenic factors (such as pelvic surgery, radiotherapy or chemotherapy, uterine artery embolism, drugs such as hormones or immunosuppressants); (3) participants who have taken hormone drugs in recent 3 months, such as estrogen, contraceptive, and glucocorticoids; (4) the score of Self-Rating Anxiety Scale (SAS) greater than 70; and (5) serious cardiovascular, cerebral, liver or kidney diseases, cancer, or psychiatric disease.

### Randomization and blinding

This trial will use the central randomization system provided by the Institute of Basic Research in Clinical Medicine of CACMS. Eligible participants will be randomly assigned to acupuncture or control groups in a 1:1 ratio. The randomization scheme and its related parameters protected by a strict viewing permission will be kept as blind code by a member of staff who is not involved in this study. The allocation sequence will be computer-generated. Acupuncturists will be responsible for randomization. They will obtain a random number and group assignment after entering gender and birthday of eligible participants into the central randomization system over cellphone or web. Participants, outcome assessors, and statisticians will be blind to treatment allocation during the whole study. Acupuncturists will not take part in the outcome assessment. Emergency unblinding is generally not required when serious adverse event (SAE) occurs. Unblinding during the trial will be permissible when acupuncture may affect the choice of treatment for SAE.

### Interventions

In this trial, *Hwato* disposable needles of different specifications (0.25 × 25 mm, 0.25 × 40 mm, and 0.30 × 75 mm) and SDZ-III electroacupuncture (EA) apparatus (Suzhou Medical Appliance, Jiangsu, China) will be used. Interventions in both groups will be administered by three acupuncturists who are registered practitioners of traditional Chinese medicine (TCM) and have at least 3 years’ clinical experience in acupuncture practice. Acupuncturists will receive training on study protocol and standard acupuncture manipulation before study initiation.

### Acupuncture group

The protocol for this group is based on clinical experience of the corresponding author [18]. It consists of two groups of acupoints: group 1 (in supine position), GV20 (Baihui), GV24 (Shenting), GB13 (Benshen), CV12 (Zhongwan), ST25 (Tianshu), CV4 (Guanyuan), KI12 (Dahe), EX-CA1 (Zigong), SP6 (Sanyinjiao), and LR3 (Taichong), and group 2 (in prone position), BL23 (Shenshu), BL33 (Zhongliao), and KI3 (Taixi). All the acupoints will be located according to the “WHO Standard Acupuncture Point Locations in the Western Pacific Region” [19]. These two groups of acupoints will be used alternatively with acupoints of group 1 as initial treatment. GV20, GV24, and GB13 will be needled with transverse insertion for 10 to 20 mm. LR3 will be inserted towards the direction of KI1 (Yongquan) for 10 to 20 mm. BL33 will be needled deeply into the third posterior sacral foramina for 60 to 70 mm. Other acupoints will be needled perpendicularly for 30 to 40 mm. Each acupoint is required to obtain deqi sensation (soreness, numbness, distention, heaviness, etc.), and if needed, needle manipulation techniques (like lifting, thrusting, or twirling the needle, etc.) can be applied to promote qi arrival. For acupoints of group 2, paired alligator clips from the EA apparatus will be connected to homolateral BL23 and BL33 with a dilatational wave and a current intensity that patients can tolerate. Participants will receive 3 sessions of acupuncture treatment per week (ideally every other day) for 12 weeks. Each acupuncture session will last for 20 min.

### Control group

In this trial, we will use superficial acupuncture in the same acupoints with the acupuncture group as sham control. All acupoints in this group will be needled into skin for approximately 1 to 3 mm with transverse insertion for GV20, GV24, and GB13 and perpendicular insertion for other acupoints. No manipulation will be administered after needle insertion to avoid deqi. When acupuncturing on the acupoints of group 2, participants will be informed to receive minor EA stimulation. The EA parameters will be the same as in acupuncture group, but electricity output will be the smallest possible one that participant agrees, and the final output should not induce visible needle shivering. The duration and frequency of the control group will be the same as in the acupuncture group.

### Permitted and prohibited concomitant treatments

All participants will be treated individually and separately from other participants to prevent communication between groups. Participants will be prohibited from taking any forms of medicine for DOR, such as sexual hormones, contraceptives, Chinese herbs, and Chinese patent medicine. Participants will be discouraged to start an IVF cycle during intervention period. Nonetheless, after completing the first 4 weeks of the intervention period, participants are allowed to terminate this trial for the initiation of an IVF cycle if required. For treatment or IVF cycles that participants have already taken, relevant information will be documented in detail.

### Discontinuation of the intervention

The intervention will be terminated when (1) SAE or pregnancy occurs and (2) participants ask to quit the trial or withdraw their informed consents.

### Measurements

#### Outcome measures

The primary outcome is the change of AFC from baseline to week 12. The differences between groups will be assessed. AFC is the number of visible ovarian follicles with a mean diameter of 2–10 mm observed during transvaginal ultrasound performed on menstrual cycle days 2–5. The number of antral follicles correlates with quantity of remaining follicles and with the ovarian response during COS. A low AFC is associated with poor response to ovarian stimulation during IVF.

Secondary outcomes are listed as follows.

(1) The mean change from the baseline in serum basal FSH, LH, and E2 will be compared between groups at week 12 and week 24.

Basal serum levels of FSH, LH, and E2 are measured on days 2–4 of the menstrual cycle. Basal FSH is commonly used to assess ovarian reserve, and high values greater than 10 IU/L are related with DOR and poor response to ovarian stimulation. An elevated basal estradiol level (greater than 50 pg/ml) in the early follicular phase indicates reproductive aging and hastened oocyte development.

(2) The mean change from baseline in serum AMH will be compared between groups at week 12 and week 24.

AMH is produced by the granulosa cells of primary, preantral, and antral follicles of 2–6 mm in diameter. It is a good predictor of oocyte quantity. A decrease of AMH indicates oocyte depletion. Since the level of AMH is fairly stable within and between menstrual cycles, it can be measured on any day of the cycle.

(3) The mean change from baseline in AFC at week 24 will be compared between groups.

(4) Change in the length of menstrual cycle at weeks 12 and 24. The length of menstrual cycle is divided into 3 categories: normal, antedated, or delayed menorrhea. The proportion of each category will be compared between groups at weeks 12 and 24.

(5) The mean change from baseline in the score of Self-Rating Anxiety Scale (SAS) will be compared between groups at weeks 12 and 24.

SAS is a self-rating scale for measuring the presence and severity of anxiety [20]. It consists of 20 items with 4 possible responses: (1) never, (2) rarely/sometimes, (3) frequently, and (4) always. The standard score is the integer part of the raw score (the sum of the 20 items) multiplied by 1.25. A higher score denotes more serious anxiety symptoms. In this trial, we will use a validated Chinese version of SAS [21].

#### Adverse events

All participants will be requested to report adverse events (AEs), i.e., any occurrence or worsening of an undesirable sign, symptom, or disease, whether or not related to acupuncture treatment. Acupuncturists will record AEs in detail including date, type, severity, measures taken by researchers, and causal relationship with the treatment. Common acupuncture-related AEs include intolerable needling pain, hematoma, and local infection.

### Statistical considerations

#### Sample size

The results of our previous study on acupuncture for DOR [22] showed that the increase in mean AFC from baseline to week 12 was 2.25 in acupuncture group and 0.30 in the wait list control. Based on these results, we assumed an increase of 0.8 in AFC for sham acupuncture after 12-week treatment. A sample size of 50 participants per group will be needed to provide 90% power and a two-sided significance level of 5%, assuming a standard deviation of 2.22. Allowing for a 20% dropout rate, 120 participants will be recruited with 60 participants per group.

#### Statistical analysis

SPSS version 20.0 (SPSS Inc., Chicago, USA) will be used for statistical analysis. According to the intention-to-treat (ITT) principle, statistical analysis will be conducted on all randomized subjects. Missing data will be imputed using multiple imputation method. Continuous data will be presented with mean and standard deviation, or median and interquartile range, while categorical data will be presented with number and percentage. For comparison with the baseline, a paired t-test or non-parametric test will be used for continuous data, and nonparametric test for categorical data. Comparisons between groups will be analyzed using the independent *t* test or non-parametric test for continuous data, chi-square test or Fisher exact test for categorical data, and nonparametric test for ranked data. All statistical tests will be two-sided, and *p* < 0.05 will be considered statistically significant.

#### Data management and quality control

The trial protocol has been repeatedly discussed by experts on acupuncture, gynecology, and reproductive medicine. Before the start of this study, all staff will be required to attend a series of training sessions. These sessions will ensure that the staff involved in this trial fully understand the study protocol, study flow, individual responsibilities, standard operating procedures for acupuncture manipulation, central randomization, data management system, etc.

This trial is performed and coordinated at the Acupuncture and Moxibustion Hospital of China Academy of Chinese Medical Sciences. As a single-center study with known minimal risks, there will not be a trial steering committee or data monitoring committee. To ensure the trial quality, the study team will meet every 4 weeks to discuss the trial progress, problems encountered, and their solutions. Routine management of the trial is performed as follows. Principle investigator YGF has overall supervision of the trial. Data quality control is performed by HSY and HFX, mainly to verify the accuracy, completeness, and timeliness of CRF data and eligibility of patients and whether implementation of acupuncture conforms to the trial protocol, etc. Participant recruitment and data entry is performed by YQY and MZH; double data entry will be applied in this trial to ensure the accuracy of data. Acupuncture treatment is performed by YGF, HFX, and LY. Outcome assessment is performed by CSZ, responsible for the collection of trial data (baseline data, CRF data, and other data) accurately and promptly. For participants who discontinue or deviate from the protocol, outcome assessor will record reasons and relevant outcome data as much as possible via returning visit, telephone, or WeChat follow-up. Preparation of investigators brochure and CRFs is performed by HSY. Preparation of protocol and revisions is performed by HFX and MZH. The audit of the trial will be done by the Clinical Evaluation Center of the China Academy of Chinese Medical Sciences at the beginning, middle, and end of the trial. Regular reminders via WeChat will be used to improve participant compliance. There are no planned additional studies using data generated in this trial.

#### Dissemination

The results of this trial will be published in its entirety in peer-reviewed journals regardless of favorable or unfavorable findings. If participants choose to be informed of the trial results, they will receive a plain summary of results via WeChat. No professional writers will be used in this trial. Authorship on future trial publications will be determined according to the author’s contribution.

## Discussion

This trial is to reveal whether acupuncture is effective in improving ovarian reserve for women with DOR. Though observational studies [23] showed promising hormone changes and improvement of TCM symptoms, RCTs focusing on women of DOR is scarce. Available RCTs on DOR are limited by small sample sizes, heterogeneity among acupuncture protocols and control methods (usually drug control or wait list), and single ovarian reserve tests (usually sex hormones) [24, 25]. In this trial, we will use superficial acupuncture as sham control. Although placebo is a better control to investigate the effect of acupuncture than superficial acupuncture, it is hard to conduct owing to the high recognition of acupuncture by patients in China and the complexity of acupuncture protocol in this trial. Given patients’ strong desire and urgent need for treatment, superficial acupuncture seems to be a better control method than placebo or wait list; nevertheless, its effect cannot be ignored.

In assessment of ovarian reserve, basal levels of FSH, LH, and E2 are the most commonly used outcomes for DOR. According to the committee opinion of the American Society for Reproductive Medicine, there is emerging evidence to support the use of AMH and AFC as a screening test for poor ovarian response [26]. AMH, produced by granulosa cells of early follicles, reflects the size of the primordial oocyte pool. It is gonadotropin-independent and thus remains relatively consistent within and between menstrual cycles. AFC correlates with both number of remaining follicles and ovarian response during COS. Compared with basal serum levels of FSH, LH, and E2, AMH and AFC are more significant predictors of poor ovarian response. Since no single measure of ovarian reserve has 100% sensitivity and specificity to detect DOR, biochemical and imaging measures (including FSH, LH and E2, AMH, and AFC) will be combined in this trial to assess the change of ovarian reserve.

This trial has several limitations. Sham acupuncture instead of placebo is used as control, which may increase the probability of false negative results. Needling method of BL33 (a deep needling into the third posterior sacral foramina) used in acupuncture group is difficult to grasp for acupuncturists without technical training. Although most of the participants enter the trial for fertility, this trial will focus on the evaluation of ovarian reserve. Live birth rate will not be assessed due to limitation of complicated influencing factors and short observation period.

In conclusion, the results of this trial will show the effect of acupuncture versus sham acupuncture in improving ovarian reserve for women with DOR.

## Trial status

Inclusion of the first patient was on 3 November 2017. Data collection is in progress. Trial is open for further inclusion. The trial ends at 3 years after inclusion of the last patient (protocol version V1.0-20170413).

## Data Availability

The data generated in the trial are available from the corresponding author on reasonable request through email.

## Abbreviations

DOR: Diminished ovarian reserve
COH: Controlled ovarian hyperstimulation
IVF: In vitro fertilization
ART: Assisted reproductive technology
IVF-ET: In vitro fertilization-embryo transfer
POI: Premature ovarian insufficiency
FSH: Follicle stimulating hormone
LH: Luteinizing hormone
AMH: Anti-Müllerian hormone
E2: Estradiol
AFC: Antral follicle count
RCT: Randomized controlled trial
SAS: Self-Rating Anxiety Scale
AEs: Adverse events
